# A Mild Traumatic Brain Injury in Mice Produces Lasting Deficits in Brain Metabolism

**DOI:** 10.1089/neu.2018.5663

**Published:** 2018-10-01

**Authors:** Danielle N. Lyons, Hemendra Vekaria, Teresa Macheda, Vikas Bakshi, David K. Powell, Brian T. Gold, Ai-Ling Lin, Patrick G. Sullivan, Adam D. Bachstetter

**Affiliations:** ^1^ Spinal Cord & Brain Injury Research Center, University of Kentucky, Lexington Kentucky.; ^2^ Department of Neuroscience, University of Kentucky, Lexington Kentucky.; ^3^ Department of Biomedical Engineering, University of Kentucky, Lexington Kentucky.; ^4^ Sanders-Brown Center on Aging, University of Kentucky, Lexington Kentucky.; ^5^ Department of Pharmacology and Nutritional Sciences, University of Kentucky, Lexington Kentucky.

**Keywords:** arterial spin labeling, biomarkers, concussion, magnetic resonance spectroscopy, mitochondria

## Abstract

Metabolic uncoupling has been well-characterized during the first minutes-to-days after a traumatic brain injury (TBI), yet mitochondrial bioenergetics during the weeks-to-months after a brain injury is poorly defined, particularly after a mild TBI. We hypothesized that a closed head injury (CHI) would be associated with deficits in mitochondrial bioenergetics at one month after the injury. A significant decrease in state-III (adenosine triphosphate production) and state-V (complex-I) driven mitochondrial respiration was found at one month post-injury in adult C57Bl/6J mice. Isolation of synaptic mitochondria demonstrated that the deficit in state-III and state-V was primarily neuronal. Injured mice had a temporally consistent deficit in memory recall at one month post-injury. Using proton magnetic resonance spectroscopy (^1^H MRS) at 7-Tesla, we found significant decreases in phosphocreatine, N-Acetylaspartic acid, and total choline. We also found regional variations in cerebral blood flow, including both hypo- and hyperperfusion, as measured by a pseudocontinuous arterial spin labeling MR sequence. Our results highlight a chronic deficit in mitochondrial bioenergetics associated with a CHI that may lead toward a novel approach for neurorestoration after a mild TBI. MRS provides a potential biomarker for assessing the efficacy of candidate treatments targeted at improving mitochondrial bioenergetics.

## Introduction

Mitochondria are essential for neuronal health and synaptic function. Mitochondrial dysfunction is well-described during the minutes-to-days after a traumatic brain injury (TBI), but there is a critical knowledge gap concerning mitochondrial bioenergetic deficits beyond the acute period after a brain injury. Substantial evidence supports the involvement of mitochondria in the neurological sequelae of acquired brain injuries and neurodegenerative diseases.^1,2^ Mitochondria isolated from pre-synaptic nerve terminals have been shown to be sensitive uniquely to the damaging effects of a TBI.^3^ A clinical history of TBI is known to increase the risk of neurodegenerative disease and dementia.^4,5^

The commonalities of mitochondrial dysfunction after TBI and in neurodegenerative diseases suggest a potential mechanism whereby TBI induces a premature aging of mitochondria—particularly synaptic mitochondria. Using a mouse model of mild TBI, induced by a closed head injury (CHI), we found strong support for our hypothesis that a mild TBI would result in a premature aging of synaptic mitochondria. Specifically, we found a decrease in mitochondrial bioenergetics at one month post-injury, particularly in neuronal synaptic mitochondrial bioenergetics, which corresponded temporally with deficits in spatial memory as measured in the radial arm water maze.

To extend the translational potential of our findings, we tested in our mouse mild TBI model two surrogate radiological measures for bioenergetics, which could be used in head-injured patients. The field of neuroimaging is far more mature in humans than in rodent models. Multi-modal magnetic resonance (MR) changes in humans after a mild TBI have been well-described,^6^ while there is a paucity of studies in mild TBI models in rodents. Technological improvements, such as high-field strength scanners (7–11 Tesla), are providing the spatial and/or temporal resolution necessary to image the rodent brain after a mild TBI.

We sought to reverse translate two advanced neuroimaging modalities, which have shown utility clinically in mild TBI^7–11^ to a mouse model of mild TBI. The MR methods included pseudo-continuous arterial spin labeling (pCASL) to measure cerebral blood flow and proton magnetic resonance spectroscopy (^1^H-MRS). The current results demonstrate that ^1^H-MRS deficits in phosphocreatine, a surrogate radiological biomarker of mitochondrial dysfunction, occur at the same chronic time point where deficits in mitochondrial bioenergetics were confirmed in isolated mitochondria. Our results provide strong support for a novel mechanism of chronic mitochondrial deficits after TBI and a translationally relevant radiological method to monitor this mechanism.

## Methods

### Animals

All procedures were approved by the Institutional Animal Care and Use Committee of the University of Kentucky, and that were conducted in accordance with the standards of proper experimentation in the Guide for the Care and Use of Laboratory Animals and ARRIVE guidelines. Experiments used four-month-old C57BL/6J mice and included an equal ratio of male and female for all end points. This project used a total of 204 mice: 36 mice were used for mitochondrial isolation; 19 mice were used for behavioral assays; 37 mice were used for pCASL and ^1^H-MRS experiment 1; 112 mice were used for ^1^H-MRS experiment 2; 22 of the 204 mice were not included in the data analysis for reasons described in the following method sections for each end point. The final number mice used for each end point are indicated in the figure or figure legend.

Mice were randomized and assigned groups before the start of the experiment. The study was completed with multiple batches of mice using a block experimental design, with each batch including all experimental groups; the order of the group was randomized for each block, so that the order of sham and CHI surgery varied for each cage. Each cage of mice included more than one experimental group. Each subject was given a unique identification number, which does not identify the experimental group. The persons conducting the mitochondrial bioenergetics (HV), behavior (TM), and neuroimaging (DNL, VB) were blinded to the treatment conditions and were not involved on the surgery or postoperative care of the mice, which could potentially lead to unblinding of the experimental groups.

### CHI

The CHI was performed as described previously.^12,13^ Briefly, mice were anesthetized with isoflurane (3–5%). The head was stabilized in a astereotaxic frame before a midline sagittal incision was made. A 1 mL latex pipette bulb filled with water was placed under the head. The stereotaxic electromagnetic impactor^14^ used a 5.0 mm flat steel tip (Leica Biosystems). It delivered a closed-skull midline impact (coordinates: mediolateral, 0.0 mm; anteroposterior, 1.5 mm) 1.0 mm deep with a controlled velocity (5.0 ± 0.2 m/sec) and a dwell time of (100 msec). No mice were eliminated because of surgery complications. Mice were randomized to a group (sham or injured), with each group containing approximately 50% males and females. Sham mice received the incision, but not the impact and were included for the three day post-injury and 28 day post-injury time points.

### Immunohistochemistry (IHC)

Mice were deep anesthetized with 5% isoflurane before transcardial perfusion with ice-cold phosphate buffered saline (PBS) for 5 min. The brains were rapidly removed, dissected, processed for IHC end points as described previously.^12,13^ Primary antibodies used included: rabbit anti-βAPP (LifeTechnologies cat# 51–2700 (1:2000)) and rabbit anti-glial fibrillary acidic protein (GFAP) (Dako Cat# Z0334; (1:10,000). Aperio ScanScope XT digital slidescanner was used to image the entire stained slide at 20x magnification to create a single high-resolution digital image.

### Mitochondrial bioenergetics

Total mitochondria were isolated from the cortex and hippocampus as described previously.^15,16^ Synaptic and nonsynaptic mitochondria were isolated from the cortex as described previously.^3^ Mitochondrial bioenergetics were measured using the Seahorse XF-24 Flux Analyzer as described previously.^16^ One mitochondria sample from the cortex was lost because of technical error.

### Six-arm radial arm water maze

A modified four-day version of the previously reported radial arm water maze (RAWM)^13,17^ was used as described previously.^12,13^ The RAWM protocol consisted of four days of training, which occurred on nonconsecutive days (that is, day 14, 15, 16, and 21 post-injury). The total trials/day were 15, and each trial was up to 60 sec long. EthoVision (Noldus Information Technology) was used for video recording and scoring behavior. A memory retention 60 sec probe trial was performed seven days after the last training day (28 days post-injury). Time spent in the goal arm and the percentage of entries in the goal were recorded.

### MR imaging

MR imaging was performed on a 7-Tesla Clinscan scanner (Bruker, Billerica, MA) using a 2 × 2 Bruker brain surface coil or a Bruker quadrature, cryocoil at the University of Kentucky Magnetic Resonance Imaging and Spectroscopy Center, as described previously.^18^ Heart rate (90–130 bpm), respiration rate, and rectal temperature (37 ± 0.5°C) were monitored continuously. A water bath with circulating water at 45–50°C was used to maintain the body temperature. The order of mice scanned was randomized between sham and CHI groups for each acquisition period.

T2-weighted structural images were acquired with field of view (FOV) = 18 × 18 mm^2^, matrix = 256 × 256; slice thickness = 1 mm, six slices, repetition time (TR) = 1500 msec, and echo time (TE) = 35 msec.

Quantitative cerebral blood flow (CBF) (units of mL/g per min) was measured using MR based pCASL. A whole-body volume coil was used for transmission, and a mouse brain surface coil was placed on top of the head for receiving. Paired control and label images were acquired with a train of Hanning window-shaped radiofrequency pulses of duration and spacing 200/200 μsec and the following parameters: flip angle = 25 degrees, slice-selective gradient = 9mT/m, labeling duration = 2100 msec, echo spacing = 0.33 msec. A two-dimensional multi-slice spin-echo planner imaging sequence was used with a FOV: 18.0 × 18.0mm^2^, matrix = 128 × 128, slice thickness = 1.0 mm, six slices, TR = 4000 msec, and TE = 20 msec with 1200 repetitions.^19^ Manual shimming was applied after automatic shimming with the lowest possible frequency obtained before the image was acquired. Ten measurements were obtained before the usable 120 measurements as a test run to optimize the phase adjustment between two successive tagging radiofrequency pulses.

^1^H-MRS was acquired in the first setup experiments using the 2 × 2-surface coil, a single ^1^H-voxel of interest was defined as the bilateral dorsal hippocampus with a size of 12.48 mm^3^ (2.0 mm × 5.23 mm × 1.2 mm). Acquisition parameters for applied PRESS spectroscopy sequence include: TR = 1500 msec, TE = 135 msec, flip angle (FA) = 90 deg, rotation (R = 0 deg), spectral width (SW = 60 Hz), and averages (A = 400). Manual shimming was applied after automatic shimming on a shimming volume 30% larger than the spectroscopy voxel to maximize the suppression of outer-voxel water. A second acquisition with 10 averages and no water suppression was acquired and used to calculate metabolite concentrations relative to water.

^1^H-MRS was acquired in the second set of experiments using the cryocoil; acquisition parameters for the LASER spectroscopy sequence^20^ for the cryocoil include: TR = 2500 msec, TE = 21 msec, FA = 90, R = 0 deg, SW = 250, and A = 200. The LASER sequence was used for the second set of experiments to maximize the suppression of outer-voxel water and use a short TE. This maximizes signal to noise, allows for the measurement of metabolites not observable at higher TEs, and increases sensitivity to metabolite concentrations. The single ^1^H-voxel of interest was defined as the bilateral dorsal hippocampus. Manual shimming was applied after automatic shimming on a shimming volume 30% larger than the spectroscopy voxel to maximize the suppression of outer-voxel water. The lowest possible full width half maximum (FWHM) was obtained, and any spectra with a FWHM greater than 40 Hz as determined by the LCmodel processing software was eliminated. A second acquisition with 10 averages and no water suppression was acquired and used to calculate metabolite concentrations relative to water.

### pCASL data analysis

MANGO software (version 3.8, UT Health, San Antonio, TX) was used to quantify the blood perfusion to the brain. pCASL images were overlaid on top of the T2-weighted structural images in MANGO software to visualize the regions of interest (ROI). Labeling efficiency (e.g., >0.85) was used to verify the quality of the image series. Four mice were excluded because of a poorly acquired image. The pCASL images were analyzed using a custom, in-house developed MATLAB (Natick, MA) script, based on published equations.^21^ A single image slice was used for the CBF quantification with approximate coordinates of 1.5–1.7 mm anterposterior).

### ^1^H-MRS data analysis

The concentrations of the metabolites were derived from the Linear Combination model (LCModel) on a Linux operation system.^22,23^ The concentrations of the metabolites (in μM) were computed using the following equation:

\begin{align*}
\left[ m \right] = \left( { { \frac { { S_m } }  { { S_ { water } } } } } \right) \left[ { water } \right] { C_n } { C_ { av } } 
\end{align*}

where [*m*] is the concentration of the specific metabolite, S_m_ is the metabolite intensity obtained from ^1^H-MRS, S_water_ is the water intensity, [water] is the water concentration (55.14 mM at 310 K), C_n_ is the correction for the number of equivalent nuclei for each resonance, and C_av_ is the correction for the number of average.^18,24^ For each spectra, a signal to noise ratio was calculated by LCModel; the FWHM estimate of 0.049 was used as a cutoff for the cyrocoil ^1^H-MRS results. Nine mice were excluded because of a FWHM greater than 0.049.

### Statistical analysis

JMP Software version 12.0 (SAS institute, Cary, NC) was used for statistical analysis. A repeated-measures analysis of variance (ANOVA) was used for RAWM. For all other end points, a one-way ANOVA was used comparing injury groups. If a significant main effect was found, *post hoc* analysis was used to compare groups. The normality assumption was assessed using the Shapiro-Wilk test, and heterogeneity of variances was assessed with the Levene test. Differences between mean were considered significant at α = 0.05. Graphs were generated using GraphPad Prism version 7.0. Values are expressed as mean ± standard error of the mean (SEM), unless otherwise noted. Scatter plots represent individual mice. No difference was found between the sham group three days post-surgery compared with the sham group at 28 days post-surgery; therefore, these groups were collapsed into a single sham group for all end points. Number of mice used for each end point are indicated in the figure or figure legend.

## Results

**FIG. 1.**
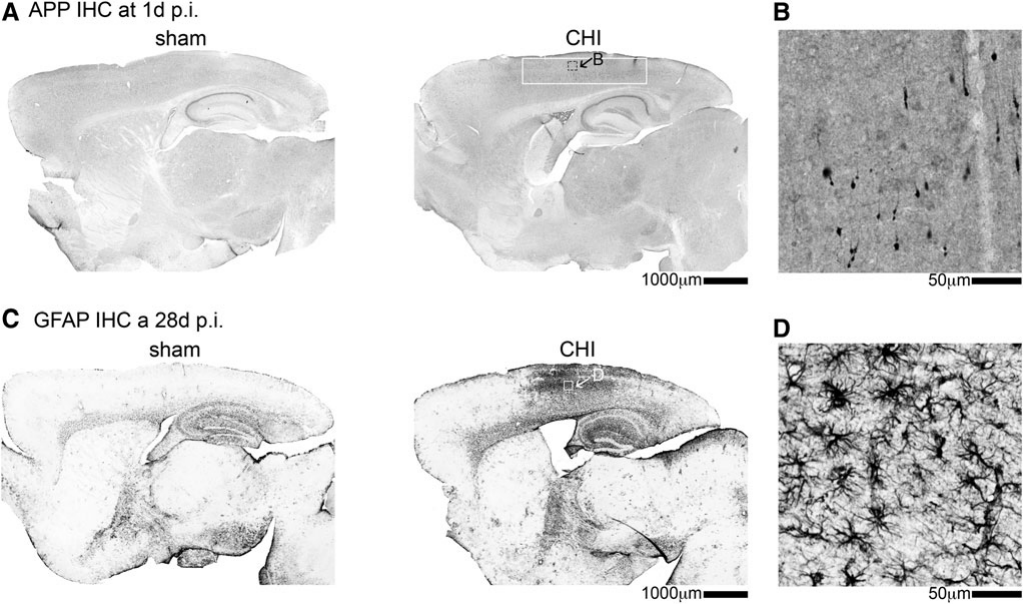
Closed head injury (CHI) causes axonal injury and astrogliosis but no structural lesions. (**A**) Area of amyloid precursor protein (APP)-positive staining is highlighted by the white box in the low magnification view in the CHI mice one day post-injury. A high magnification view of the APP staining in (A) indicated by the black dashed box is shown in (**B**). (**C**) Glial fibrillary acidic protein (GFAP) staining shows diffuse reactive astrocytes in the CHI mice at 28 days post-injury. A high magnification view of the GFAP staining in (C) indicated by the white box is shown in (**D**). IHC, immunohistochemistry.

At least 75% of the 1.7 million TBIs that occur in the United States each year are classified as a mild TBI.^25,26^ In the present study, we used a CHI to model mild TBI. As reported previously,^12,13^ and confirmed here (Fig. 1), the CHI model does not cause gross structural lesions to the brain. At a one day post-injury time point, we find markers of axonal injury in the neocortex (Fig. 1A, B). At 28 days post-CHI, we find diffuse astrogliosis (Fig. 1C, D), with the highest GFAP staining corresponding to the same region of the brain with the greatest amyloid precursor protein (APP) staining at one day post-CHI.

### C57Bl/6J mice show persistent deficits in mitochondrial bioenergetics after a mild TBI

**FIG. 2.**
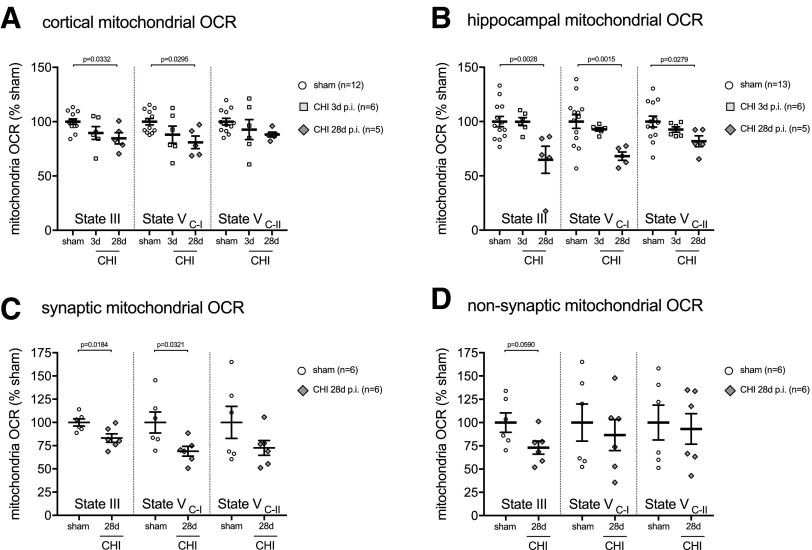
A mild traumatic brain injury (TBI) results in persistent deficits in mitochondrial bioenergetics. (**A**) Total mitochondria were isolated from the cortex of C57BL/6J mice. State III (F_2,22_ = 4.034; *p* = 0.034) and State V complex I (V _C-I_) (F_2,22_ = 4.017; *p* = 0.036) mitochondrial oxygen consumption rate (OCR) were found to be suppressed after a closed head injury (CHI). The Dunnett test showed a decrease in State III (*p* = 0.0332) and State V _C-I_ (p = 0.0295) in the 28 days post-CHI group compared with sham mice. (**B**) Hippocampal total mitochondria were found to have suppressed State III (F_2,26_ = 6.945; *p* = 0.042), State V _C-I_ (F_2,26_ = 7.40; *p* = 0.003), and State V _C-I_ (F_2,26_ = 3.602; *p* = 0.043). The Dunnett test found a decrease in State III (*p* = 0.0028), State V _C-I_ (*p* = 0.0015) and State V _C-II_ (*p* = 0.0279) in the 28 days post-CHI group compared with sham mice. (**C**) Differential isolation of neuronal presynaptic mitochondria, and (**D**) nonsynaptic mitochondria (neuronal cell bodies, glia, and infiltrating immune cells) was performed in the cortex of a second cohort of C57BL/6J mice. (C) CHI-induced a suppression in State III (T_10_ = 2.81; *p* = 0.018), and State V _C-I_ (T_10_ = 2.49; *p* = 0.032) mitochondria OCR. (D) A decrease in State III mitochondrial OCR (T_10_ = 2.13; *p* = 0.059) was found in the nonsynaptic mitochondria fraction.

Total mitochondria were isolated from the cortex and hippocampus, and State-III (adenosine triphosphate [ATP] synthesis capacity), State-V_C1_ (complex I driven maximal electron transport), and State-V_C2_ (complex II driven maximal electron transport) were measured using the Seahorse Extracellular Flux Analyzer. In the cortex, we found a stepwise decrease with time post-injury in both State-III and State-V_C-I_ mitochondrial respiration after CHI compared with the sham mice. No statistical significant difference in mitochondrial bioenergetics was found at three day post-CHI compared with sham-injured mice in the cortex (Fig. 2A) or hippocampus (Fig. 2B). By 28 days post-CHI, mice had a significant decrease in State-III and State-V_C-I_ mitochondrial respiration compared with the sham-injured mice (Fig. 2A). In the hippocampus, we found a decrease in State-III, State-V_C-I_, and State-V_C-II_ mitochondrial respiration at 28 days post-CHI compared with the sham-injured mice (Fig. 2B).

A brain-region heterogeneity was evident in the CHI-induced mitochondrial bioenergetics dysfunction between the hippocampus and cortex. In the hippocampus at 28 days post-CHI, the deficit in mitochondrial respiration compared with shams was 35% in State-III and 32% State-V_C-I_. In contrast, the cortex at 28 days post-CHI compared with shams was found to have a 16% deficit in State-III and 20% deficit in State-V_C-I_. It is unknown whether the regional heterogeneity in mitochondrial bioenergetics is a reflection of different sensitivity of the mitochondria in the brain regions to the effect of the injury, or differences in the degree of the primary injury in the two brain regions.

After a CHI, the cortex but not the hippocampus has profound gliosis (microglia and astrocytes) and immune cells infiltrates (macrophages and neutrophils).^12,13^ We hypothesized that the infiltration of immune cells and proliferation of glia in the cortex could mask some deficits in neuronal mitochondrial bioenergetics, compared with that seen in the hippocampus. To test this hypothesis, in a second cohort of mice, we performed differential isolation of neuronal presynaptic mitochondria and nonsynaptic mitochondria (neuronal cell bodies, glia, and infiltrating immune cells) in the cortex at 28 days post-CHI. In the synaptic mitochondria fraction, we found a 17% deficit in State-III and 31% deficit in State-V_C-I_ (Fig 2C). In the nonsynaptic, there was a 27% deficit in State-III, while State-V_C-II_ was unchanged from sham levels (Fig 2D).

### Mild TBI causes impaired hippocampal-dependent learning and memory

**FIG. 3.**
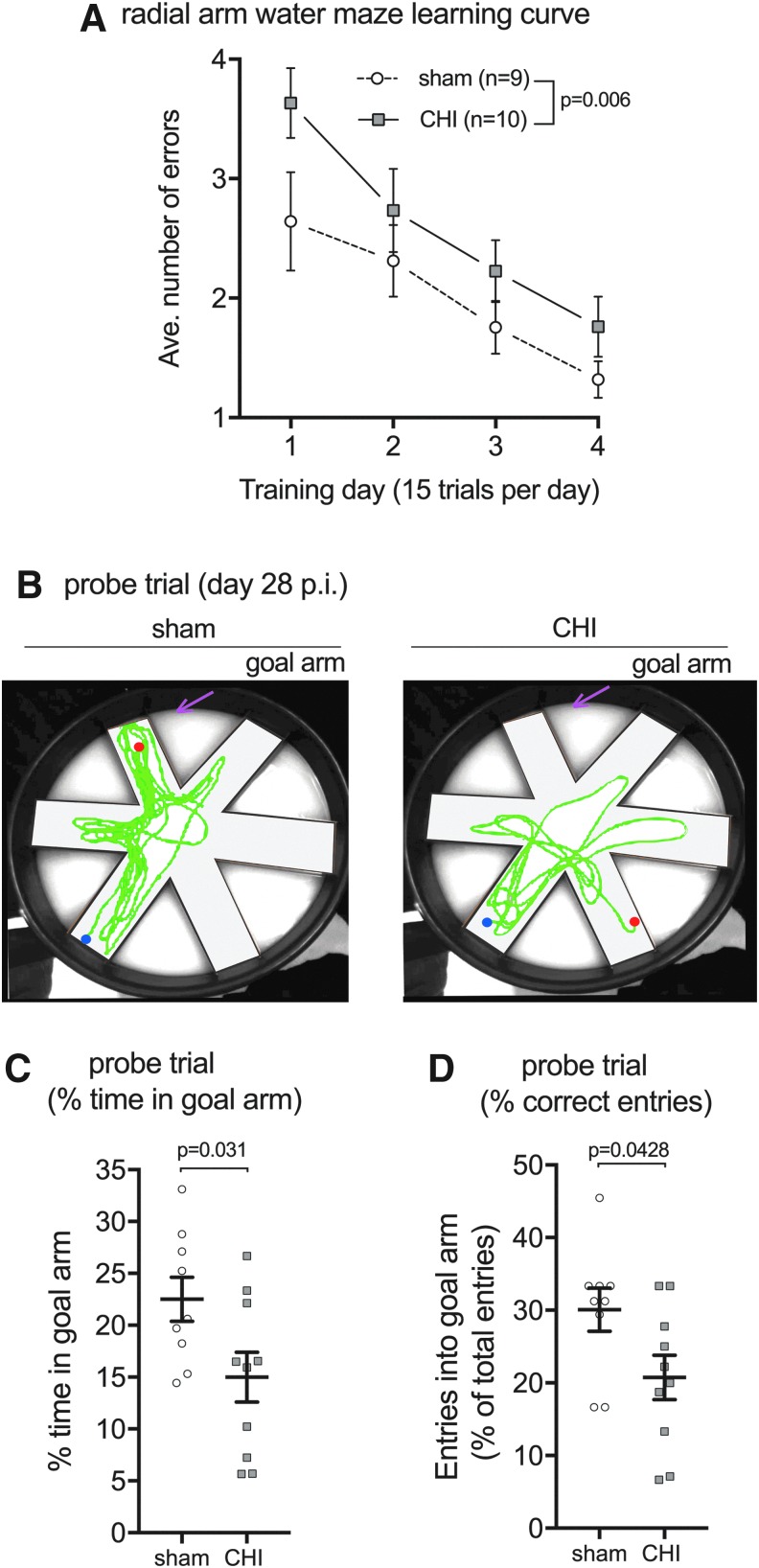
Closed head injury (CHI)-induced deficits in the radial arm water maze. (**A**) To measure CHI-induced learning deficits, mice received 15 trials a day of testing over four nonconsecutive days (that is, day 14, 15, 16, and 21 post-injury) in the six-arm radial arm water maze with day 1 of testing starting on day 14 post-injury. CHI mice made more errors in finding the escape platform compared with sham-injured mice (F = 7.977; *p* = 0.0062); however, both groups were able to learn the task. Day 1 includes both visible and hidden platform trials. Only hidden platform trials are included in the data analysis and graph for Day 1. The memory of platform location was tested by a probe trial, where the escape platform was removed. The probe trial was conducted seven days after the last training session, at day 28 post-injury. (**B**) Computer-generated trace of the animal's movements is shown for a representative sham and CHI mouse. Start position for the animal is indicated with the blue circle. Stop position for the animal is indicated with the red circle. (**C**) Sham-injured mice spent significantly more time in the goal arm, where the escape platform had been located, compared with CHI mice (T_17_ = 2.319; *p* = 0.0331). (**D**) Sham-injured mice entered the goal arm more often than the nongoal arms compared with the CHI mice (T_17_ = 2.189; *p* = 0.0428). Color image is available online at www.liebertpub.com/neu

In light of the persistent suppression of hippocampal and neuronal mitochondrial bioenergetics after a CHI, we hypothesized that injured mice would have impairments in hippocampal-dependent learning and memory. To measure the cognitive status of the injured mice, we used the six-arm RAWM test of spatial learning and memory. The CHI-injured mice were found to have a delayed learning curve over the four days of training compared with the sham-injured mice (Fig. 3A). Importantly, both groups, CHI and sham, were eventually able to find the location of the hidden platform while making less than two errors by the fourth day of training (Fig. 3A).

Long-term memory consolidation was measured by using a probe trial, where the hidden platform was removed, and the amount of time and number of correct entries into the goal arm were considered an indicator of a memory of the platform location. The probe trial was conducted seven days after the last training session, on day 28 post-injury. As shown in Figure 3B, CHI mice showed no preference for the goal arm, whereas sham-injured mice were found to have a preference for the goal arm. CHI mice spent less time in the target arm (Fig. 3C) and made fewer entries into the target arm (Fig. 3D) compared with sham-injured mice.

### Regional variations in CBF after CHI

**FIG. 4.**
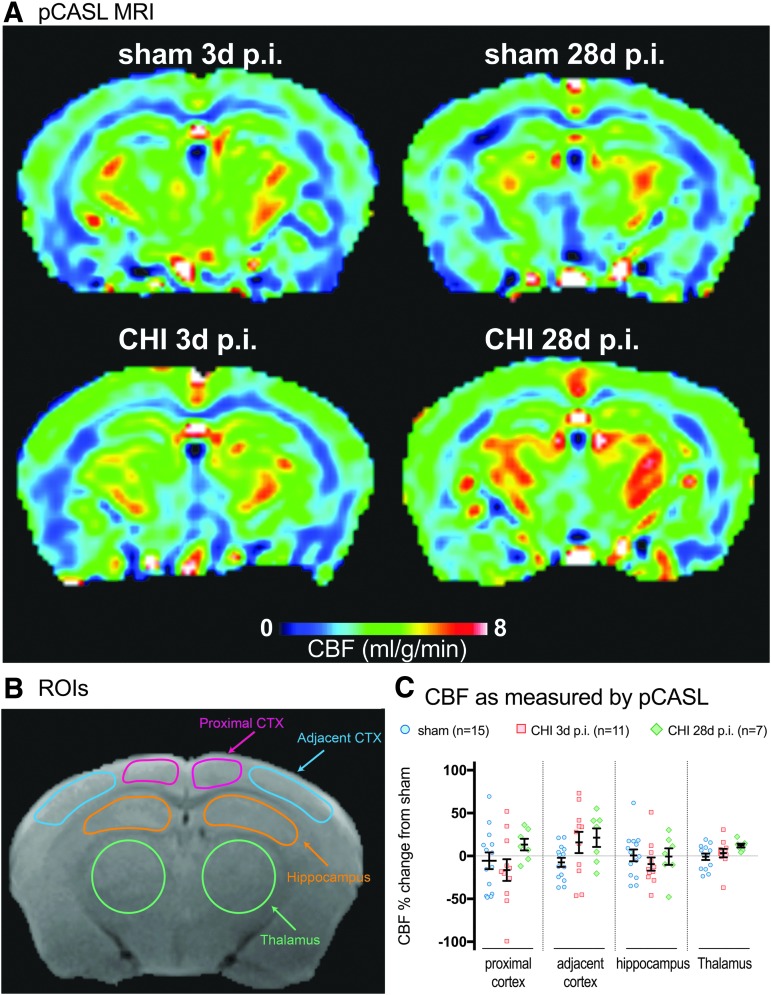
Cerebral blood flow (CBF) as measured by Pseudo-Continuous Arterial Spin Labeling (pCASL). (**A**) Representative CBF perfusion maps of sham and CHI both at three and 28 days post-injury. An increase in CBF (mg/g/min) is depicted with warmer colors and a decrease in CBF is shown by cooler colors. (**B**) Four regions of interest (ROIs) analyzed, shown on the T2 structural image, include: (i) proximal cortex (CTX) (closest to injury); (ii) adjacent cortex; (iii) hippocampus; and (iv) thalamus. **(C)** Quantification of the CBF in the four ROIs expressed as change from sham for each ROI. Color image is available online at www.liebertpub.com/neu

Multimodal neuroimaging has the potential to be used as surrogate measures of mitochondrial bioenergetics. We sought to determine in a pilot experiment whether CBF and ^1^H-MRS would be sensitive biomarkers of metabolic disturbance after CHI in mice. We chose to measure CBF and ^1^H-MRS because these two MR modalities are commonly available clinically and could be completed in one scanning session. To measure CBF, we used the pCASL method. pCASL has higher signal to noise ratio and better labeling efficiency than other arterial spin labeling techniques. Also, pCASL allows for regional analysis to look for heterogeneity in CBF.^21,27^ Examples of the CBF perfusion maps are shown in Figure 4A. To quantify the regional heterogeneity in CBF after injury, we chose to analyze four distinct ROIs: (i) proximal cortex (closest to injury); (ii) adjacent cortex; (iii) hippocampus; and (iv) thalamus (Fig. 4B). The ROIs were based on our previous studies, which defined the patterns of neuropathological changes in the CHI model.^12,13^

We observed subtle regional and temporal changes in the pattern of the CBF. Specifically, a 16.4% decrease in CBF was found in the three days post-CHI group in the region of the cortex most proximal to the site of injury compared with the sham and 28 days post-CHI group (Fig. 4C). In the cortex adjacent to the site of injury, an increase in CBF was found in the three days post-CHI group (15.8%) and 28 days post-CHI group (21.4%) compared with the sham group (Fig. 4C). Similar to the proximal cortex, a small decrease was found in CBF in the hippocampus in the three days post-CHI group (-9.4%) compared with the sham or 28 days post-CHI group (Fig. 4C). The thalamus was found to have a slight increase in CBF in the 28 days post-CHI group (12.1%) compared with the sham or three days post-CHI group (Fig. 4C).

Apparent from the scatter plots in Figure 4, a sizable amount of variability in CBF was seen within the groups. While patterns of CBF changes after a CHI are evident, none of the changes were found to be statistically significant. The amount of variability between the brain regions was relatively proportional to the size of the ROI, indicating that the smaller ROIs were pushing the limits of the scanner. A goal of the study was to determine the feasibility of using pCASL to detect CHI-induced changes in CBF. Results of *post hoc* power analysis showed that to detect an effect size of 10–20% change in CBF in the neocortex or hippocampus caused by a mild TBI would require an unrealistic number of mice per group (*n* = 144–56, respectively). The number of mice needed to detect a similar magnitude change in the thalamus was reasonable (*n* = 21). The results suggest that further improvements in the MR methods to increase the sensitivity of CBF in small ROIs may allow for MR to be used to detect changes in CBF after a mild brain injury in mice.

### A mild TBI decreases hippocampal metabolites associated with energy metabolism (PCr) and neuron function (NAA and tCho)

**FIG. 5.**
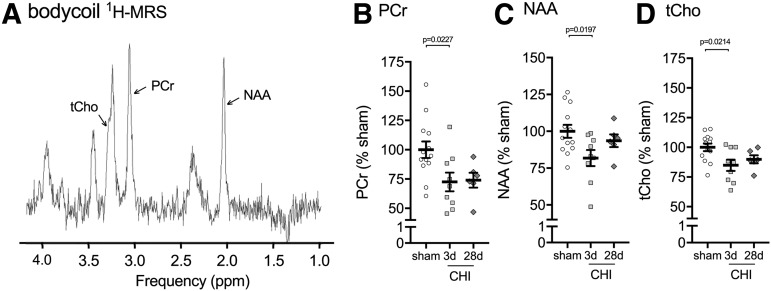
Closed head injury (CHI)-induced changes in hippocampal metabolites measured by brain surface coil ^1^H-MRS. (**A**) Representative example of the proton magnetic resonance spectroscopy (^1^H-MRS) spectrum acquired in the dorsal hippocampus of mice. A significant decrease was found in (**B**) phosphocreatine (PCr) (F_2,27_ = 4.63, *p* = 0.0195), (**C**) N-acetylaspartate (NAA) (F_2,27_ = 3.87, *p* = 0.0345), and (**D**) total choline (tCho) (glycerophosphorylcholine + phosphorylcholine) (F_2,27_ = 4.50, *p* = 0.0214) in CHI mice compared with sham-injured mice. *Post hoc* comparisons were made using a Dunnett test comparing all CHI groups with sham. Each symbol on the scatter plot represents a single animal. Sham (*n* = 13), three days post-CHI (*n* = 9), and 28 days post-CHI (*n* = 6).

The second multi-modal neuroimaging sequence acquired in the same scanning session with pCASL was ^1^H-MRS in the bilateral dorsal hippocampus using a Bruker brain surface coil. Previous studies have demonstrated reliable detection of metabolites with a voxel of interest covering the bilateral dorsal hippocampus.^18^ While we anticipated a larger change in metabolites in the cortex immediately adjacent to the site of injury, this region was not selected as the ROI because the size and shape of the region would result in a voxel of interest that we believed would be too small to measure metabolites accurately. Figure 5A shows a representative spectrum of the metabolites acquired by ^1^H-MRS in the dorsal hippocampus of a mouse.

A total of five metabolites were acquired by ^1^H-MRS: phosphocreatine (PCr), N-acetylaspartate (NAA), glutamate (Glu) taurine (Tau) and total choline (tCho). Of the five metabolites, three metabolites were found to be significantly decreased after the injury. PCr was found to be reduced in the hippocampus at three days post-CHI (-26%) and 28 days post-CHI (-27.5%) compared with the sham-injured group (Fig. 5B). NAA was reduced at three days post-CHI (-18.2%) compared with the sham-injured group (Fig. 5C). tCho was found to be reduced in the hippocampus at three days post-CHI (-15.1%) and 28 days post-CHI (-10.1%) compared with the sham-injured group (Fig. 5D). In comparison with the pCASL, power analysis on the pilot study of ^1^H-MRS determined that a reasonable sample size would be needed in future studies to detect CHI-induced changes in PCr, NAA, and tCHo by ^1^H-MRS in the dorsal hippocampus (*n* = 15, 7, 4; per group for PCr, NAA, tCho, respectively, for 20% effect size).

**FIG. 6.**
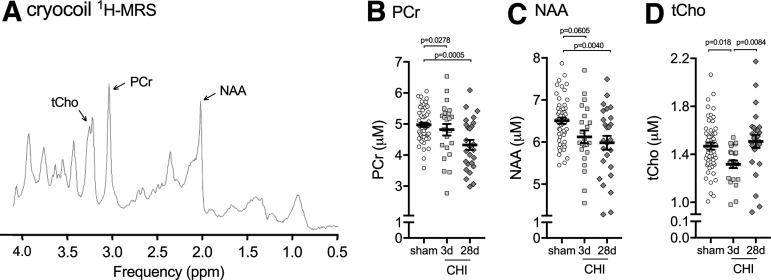
Closed head injury (CHI)-induced changes in hippocampal metabolites measured by cryocoil ^1^H-MRS. (**A**) Representative example of the proton magnetic resonance spectroscopy (^1^H-MRS) spectrum acquired in the dorsal hippocampus of mice. A significant decrease was found in (**B**) phosphocreatine (PCr) (F_2102_ = 7.569, *p* = 0.0009), (**C**) N-acetylaspartate (NAA) (F_2,102_ = 6.294, *p* = 0.0027), and (**D**) total choline (tCho) (glycerophosphorylcholine + phosphorylcholine) (F_2,102_ = 5.281, *p* = 0.0066) in CHI mice compared with sham injured mice. *Post hoc* comparisons were made using a Tukey-Kramer test comparing all groups. Each symbol on the scatter plot represents a single animal. Sham (*n* = 54), three days post-CHI (*n* = 23), and 28 days post-CHI (*n* = 26).

To confirm the changes in hippocampal metabolites found in the pilot study, we generated a second cohort of sham and CHI mice to repeat the ^1^H-MRS. To increase the signal to noise, we used a Bruker cryocoil. The cryocoil provided a 2.5-fold increase in signal to noise, which is a 6.25-fold improvement in imaging time. In addition, we used an improved water suppression sequence called LASER,^20^ which allowed us to reduce the echo time while still suppressing the outer voxel water (Fig. 6A). These improvements in cryocoil and LASER sequence increased the total number of metabolites detected from five to 18 (Table 1).

**Table 1.** Hippocampal Metabolites Detected by Cryocoil ^1^H-MRS in the Hippocampus of Mice after Sham or Closed Head Injury Surgical Procedure

	*SHAM (mean ± SD μM)* n* = 54*	*3D CHI (mean ± SD μM)* n* = 23*	*28D CHI (mean ± SD μM)* n* = 26*
Ala	1.097 ± 0.381	1.267 ± 0.455	1.073 ± 0.757
Cr	2.407 ± 0.51	2.206 ± 0.383	2.603 ± 0.89
PCr	4.975 ± 0.524	4.816 ± 0.898	4.326 ± 0.824
GABA	4.143 ± 0.598	3.753 ± 0.524	3.841 ± 0.884
Gln	3.527 ± 1.235	3.764 ± 1.375	3.593 ± 2.053
Glu	10.187 ± 1.089	9.788 ± 1.176	9.748 ± 1.583
GSH	1.295 ± 0.249	1.27 ± 0.266	1.296 ± 0.402
Ins	6.588 ± 0.847	6.127 ± 0.931	6.494 ± 1.161
Lac	3.018 ± 1.003	2.961 ± 0.836	2.415 ± 1.105
NAA	6.508 ± 0.54	6.125 ± 0.732	5.983 ± 0.841
NAAG	0.723 ± 0.369	0.658 ± 0.382	0.65 ± 0.345
Tau	9.986 ± 1.351	9.552 ± 1.534	9.629 ± 2.036
CrCH2	0.82 ± 0.398	0.962 ± 0.43	0.661 ± 0.354
tCho	1.469 ± 0.215	1.317 ± 0.153	1.508 ± 0.275
MM09	7.122 ± 1.132	6.989 ± 0.804	7.516 ± 1.456
Lip20	0.027 ± 0.127	0.053 ± 0.176	0.079 ± 0.227
MM20	8.128 ± 1.905	7.953 ± 2.762	7.947 ± 2.374
MM14	6.189 ± 2.401	5.86 ± 1.346	5.619 ± 1.64

CHI, closed head injury; SD, standard deviation; Ala, alanine; Cr, creatine; PCr, phosphocreatine; GABA, gamma-aminobutyric acid; Glu, glutamate; Gln, glutamine; GSH, glutathione; Oms, myoinositol; Lac, lactate; NAA, N-acetylaspartate; NAAG, N-acetylaspartylglutamate; tau, taurine; –CrCH2, creatine methylene group; tCHo, total choline; Lip, mobile lipids; and MM, macromolecules.

The pattern of hippocampal metabolite changes seen with the cryocoil ^1^H-MRS was in agreement with the changes seen using the brain surface coil; however, the magnitude of the changes in metabolites was smaller, which likely reflects a decrease in variability associated with greater sample size and reduced variance from the 2.5 times more signal-to-noise using the cryocoil compared with the brain surface coil. We found that PCr was decreased 3% at three days post-CHI and 13% by 28 days post-CHI compared with sham levels (Fig. 6B). NAA was decreased by 6% at three days post-CHI compared with sham levels (Fig. 6C). In contrast to the brain surface coil where NAA levels had normalized by 28 days post-CHI, we found an 8% decrease in NAA at 28 days post-CHI compared with sham levels (Fig. 6C). The most consistent changes between the brain surface coil and cryocoil ^1^H-MRS were found with tCho. A 10% decrease in tCho was found at three days post-CHI compared with sham levels (Fig. 6D), with levels of tCho returning to sham values by 28 days post-CHI.

### Cryocoil ^1^H-MRS as a radiological biomarker of TBI-induced deficits in brain metabolism and neuronal function

We next sought to determine the usefulness of cryocoil ^1^H-MRS as a surrogate indicator of deficits in brain metabolism and neuronal function. We chose to use two threshold values with varying degrees of stringency. We wanted to assess how accurately we could separate injured mice from sham mice while maintaining the robustness to identify injured mice with deficits in surrogate indicators of brain metabolism or neuronal function. One standard deviation below mean sham levels of the metabolites was used as our low stringency threshold. For high stringency, we used a threshold just below the lowest sham value.

**FIG. 7.**
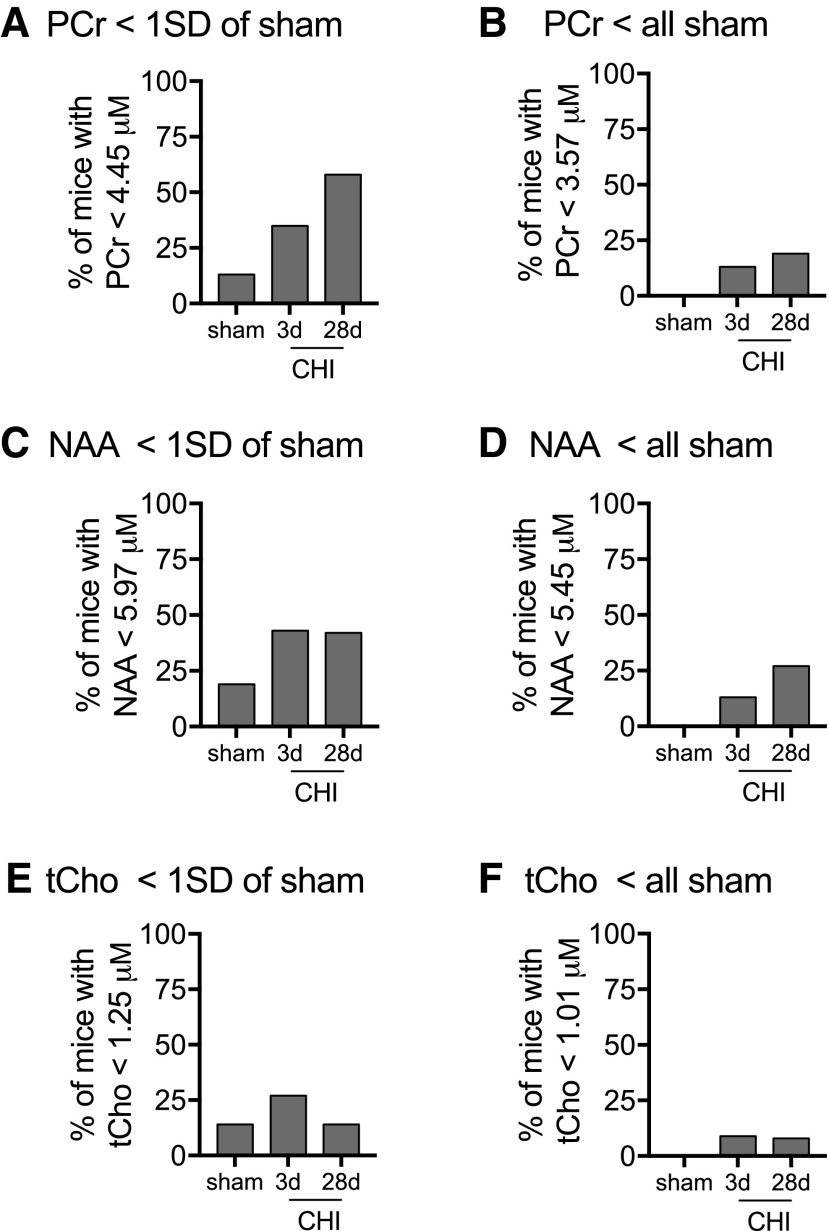
Discriminative potential of ^1^H-MRS as a surrogate indicator of deficits in brain metabolism and neuronal function. The percentage of mice with a PCr (phosphocreatine) value less than 4.44 μM (i.e., one standard deviation (SD) of sham mean levels (**A**) or below the lowest sham PCr value (3.57 μM) (**B**) is shown. The percentage of mice with a NAA value less than 5.97 μM (i.e., one SD of sham mean levels (**C**) or below the lowest sham NAA (N-acetylaspartate) value (3.57 μM) (**D**) is shown. The percentage of mice with a tCHo (total choline) value less than one SD of sham mean levels (1.26 μM) (**E**) or below less lowest sham tCho value (1.01 μM) (**F**) is shown. Sham (*n* = 54), three days post-closed head injury (CHI) (*n* = 23), and 28 days post-CHI (*n* = 26).

For PCr, the one standard deviation threshold was able identify 35–58% of the three days post-CHI and 28 days post-CHI mice, respectively; while only falsely identifying 13% of the sham-injured mice as having a deficit in PCr (Fig. 7A). With the high stringency threshold, we eliminated false detection of PCr deficits, and still maintained 13–19% of three days post-CHI and 28 days post-CHI mice, respectively, identified with deficits in PCr (Fig. 7B). NAA was able to identify 42–43% of the 28 days and three days injured mice, with a false positive rate of 19% in the sham-injured mice when a one standard deviation threshold was used (Fig. 7C). The detection rate of brain-injured mice with deficits in NAA was reduced to 13–27% of three days and 28 days post-CHI mice, respectively, when all false positives were eliminated (Fig. 7D). The discrimination potential of tCho was low, only identifying 27% of three days post-CHI mice while having a false positive rate of 14% in the sham-injured mice (Fig. 7E). The discrimination potential of tCHO to identify brain-injured mice was less than 10% when all false positives were eliminated (Fig. 7F).

### Female mice show increased vulnerability to CHI-induced changes in hippocampal metabolites

**FIG. 8.**
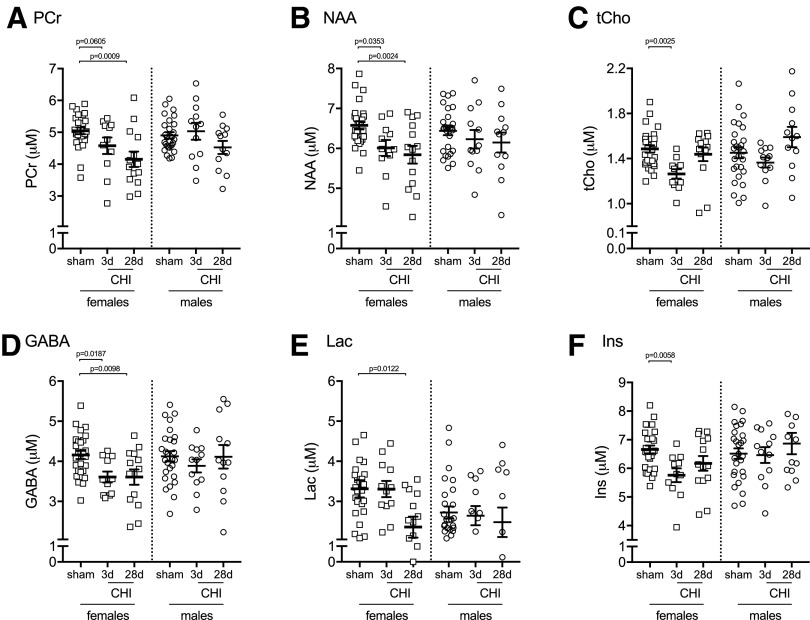
Stratification by sex identifies closed head injury (CHI)-induced changes in six hippocampal metabolites measured by cryocoil ^1^H-MRS. A significant change was found in (**A**) phosphocreatine (PCr) (F_2,51_ = 7.354, *p* = 0.0016), (**B**) N-acetylaspartate (NAA) (F_2,51_ = 6.061, *p* = 0.0044), (**C**) total choline (tCho) (glycerophosphorylcholine + phosphorylcholine) (F_2,51_ = 5.881, *p* = 0.0051), (**D**) gamma-aminobutyric acid (GABA) (F_2,51_ = 6.061, *p* = 0.0044), (**E**) lactate (Lac) (F_2,51_ = 4.511, *p* = 0.0159), and (**F**) myoinositol (Ins) (F_2,51_ = 5.295, *p* = 0.0083) in female mice. No statistically significant changes were found in male mice. *Post hoc* comparisons were made using a Dunnett test comparing all CHI groups with sham. Each symbol on the scatter plot represents a single animal. Female sham (*n* = 27), female three days post-CHI (*n* = 11), and female 28 days post-CHI (**n** = 14). Male sham (*n* = 27), male three days post-CHI (*n* = 12), and male 28 days post-CHI (*n* = 12).

To assess sources of variability that contribute to changes in hippocampal metabolites, we stratified the data by sex (Table 2). In female mice, we found six metabolites that were showing a significant effect of CHI; however, in male mice, no statistically significant changes in hippocampal metabolites were observed. The metabolites that were found to be significantly affected by the CHI included PCr (Fig. 8A), NAA (Fig. 8B), and tCho (Fig. 8C), which showed a similar injury-induced pattern of changes in both the female and male mice. In addition, three metabolites were found to be changed by the injury only in female mice, including gamma-aminobutyric acid (GABA) (Fig. 8D), lactate (Fig. 8E), and myoinositol (Fig. 8F).

**Table 2.** Hippocampal Metabolites Detected by Cryocoil ^1^H-MRS in the Hippocampus in Female versus Male Mice after Sham or Closed Head Injury Surgical Procedure

	*♀ SHAM (mean ± SD μM)* n* = 27*	*♀ 3D CHI (mean ± SD μM)* n* = 11*	*♀ 28D CHI (mean ± SD μM)* n* = 14*	*♂ SHAM (mean ± SD μM)* n* = 27*	*♂ 3D CHI (mean ± SD μM)* n* = 12*	*♂ 28D CHI (mean ± SD μM)* n* = 12*
Ala	0.88 ± 0.22	1.258 ± 0.558	1.146 ± 0.446	1.297 ± 1.069	1.276 ± 0.361	1.049 ± 0.303
Cr	2.4 ± 0.518	2.106 ± 0.41	2.299 ± 0.468	2.84 ± 1.17	2.298 ± 0.347	2.516 ± 0.535
PCr	4.154 ± 0.9	4.579 ± 0.86	5.047 ± 0.53	4.526 ± 0.71	5.033 ± 0.913	4.904 ± 0.517
GABA	3.607 ± 0.71	3.609 ± 0.455	4.159 ± 0.53	4.114 ± 1.014	3.886 ± 0.567	4.126 ± 0.668
Gln	4.069 ± 2.718	4.23 ± 1.578	3.52 ± 0.641	3.037 ± 0.47	3.337 ± 1.051	3.533 ± 1.643
Glu	9.386 ± 1.562	9.522 ± 1.155	10.288 ± 0.946	10.169 ± 1.565	10.032 ± 1.192	10.086 ± 1.225
GSH	1.166 ± 0.263	1.137 ± 0.24	1.241 ± 0.27	1.448 ± 0.488	1.392 ± 0.236	1.349 ± 0.217
Ins	6.174 ± 0.967	5.758 ± 0.797	6.662 ± 0.72	6.867 ± 1.296	6.465 ± 0.947	6.515 ± 0.965
Lac	2.36 ± 0.979	3.305 ± 0.728	3.313 ± 1.119	2.48 ± 1.278	2.645 ± 0.829	2.723 ± 0.786
NAA	5.842 ± 0.828	6.011 ± 0.668	6.578 ±H 0.531	6.148 ± 0.862	6.229 ± 0.8	6.438 ± 0.55
NAAG	0.637 ± 0.329	0.746 ± 0.398	0.797 ± 0.311	0.665 ± 0.377	0.578 ± 0.364	0.649 ± 0.411
Tau	8.818 ± 1.874	8.788 ± 1.426	9.72 ± 1.361	10.576 ± 1.86	10.253 ± 1.317	10.253 ± 1.312
CrCH2	0.671 ± 0.329	0.793 ± 0.344	0.811 ± 0.41	0.649 ± 0.396	1.117 ± 0.454	0.83 ± 0.393
tCho	1.438 ± 0.225	1.264 ± 0.138	1.486 ± 0.17	1.591 ± 0.313	1.365 ± 0.155	1.452 ± 0.255
MM09	7.14 ± 0.776	6.853 ± 1.061	7.155 ± 1.26	7.955 ± 1.927	7.113 ± 0.483	7.089 ± 1.011
Lip20	0.037 ± 0.133	0 ± 0	0.032 ± 0.156	0.128 ± 0.302	0.102 ± 0.237	0.022 ± 0.094
MM20	7.829 ± 2.693	8.78 ± 2.635	7.939 ± 1.789	8.086 ± 2.049	7.195 ± 2.762	8.317 ± 2.031
MM14	5.217 ± 1.11	5.938 ± 1.829	6.697 ± 3.108	6.088 ± 2.053	5.789 ± 0.755	5.681 ± 1.247

CHI, closed head injury; SD, standard deviation; Ala, alanine; Cr, creatine; PCr, phosphocreatine; GABA, gamma-aminobutyric acid; Glu, glutamate; Gln, glutamine; GSH, glutathione; Oms, myoinositol; Lac, lactate; NAA, N-acetylaspartate; NAAG, N-acetylaspartylglutamate; tau, taurine; –CrCH2, creatine methylene group; tCHo, total choline; Lip, mobile lipids; and MM, macromolecules.

## Discussion

Two key findings from the studies are reported here. First, lasting deficits in mitochondrial-driven energy metabolism were found after a single, comparatively mild, diffuse brain injury. The injury-induced changes in mitochondrial metabolism were strongest in the hippocampus and synaptic nerve terminals. Unexpectedly, the deficits in mitochondrial bioenergetics worsened with time. TBI is one of the most well-described risk factors for the development of dementia.^4,5,28^ The chronic effects of TBI on mitochondrial bioenergetics could be part of the mechanism contributing to the enhance risk of dementia developing, potentially associated with a premature aging of mitochondria.

The second relevant finding was the ability of MR spectroscopy to provide a way to monitor energy metabolism and neuronal health after a TBI noninvasively. Our results expand the utility of ^1^H-MRS beyond humans and large animals toward the use of ^1^H-MRS in mouse models of mild TBI. The ability to use the same efficacy biomarker in pre-clinical and clinical studies can provide a translational bridge in the important experiential medicine stage of drug development.^29^

### A mild TBI induces changes to mitochondrial bioenergetics similar to those seen with aging

The mechanisms by which a TBI hastens the onset of dementia are poorly defined. Aging is known to cause global decreases in brain metabolism, including both oxidative phosphorylation^30^ and aerobic glycolysis.^31^ Mitochondria are the primary source of oxygen radicals, and this underlies the mitochondrial free radical theory of aging.^32^ Mitochondrial enzymes are at risk of damage when the antioxidant buffering capacity does not match demand. Increases in mitochondrial oxidative damage are known to occur with age.^33^ Similarly, after TBI, elevated levels of intracellular Ca^2+^ are buffered by mitochondria, protecting the cell but potentially damaging mitochondria by the generation of oxidative stress.^34^ Mitochondrial oxidative damage after TBI preferentially targets pyruvate dehydrogenase, the gate-keeper of mitochondrial respiration and ATP production.^35^ Once pyruvate dehydrogenase is damaged, pyruvate cannot be converted into acetyl CoA, stopping the flow of electrons into the electron transport system by nicotinamide adenine dinucleotide and flavin adenine dinucleotide.

Prior work has shown that TBI suppresses mitochondrial respiration up to 72 h after the brain injury.^36^ It has been assumed that with time after TBI, the damaged mitochondria would be eliminated by mitophagy, or the damaged mitochondria would lead to the death of the host cell. Our results contradict this assumption and suggest that in the case of a mild TBI, the deficits in mitochondrial bioenergetics propagate across time. Our finding suggests that TBI leads to changes in mitochondria akin to those seen with aging. Future studies are warranted to experimentally test whether long-term mitochondrial dysfunction after a mild TBI contributes to TBI-induced deficits in cognitive function and enhanced risk for dementia, and to determine whether therapeutic interventions targeting chronic mitochondrial dysfunction could reduce that risk.

### Hippocampal mitochondria are selectively vulnerable to the effects of a mild TBI

Selective neuronal vulnerability is a hallmark of neurodegenerative diseases. Evidence suggests that neurons in general, and particularly neurons in the CA1 region of the hippocampus, have increased vulnerability to oxidative stress^37^ and have decreased ability to buffer Ca^2+^, which contributes to the greater neuronal mitochondria dysfunction after a TBI.^3,38,39^ In agreement with neuronal selective vulnerability, we found that a CHI resulted in a statistically significant suppression of mitochondrial respiration in the neuronal pre-synaptic mitochondria. Our previous results had suggested that the neocortex, most proximal to the direct forces of the CHI, was associated with the most pathology as determined by reactive gliosis.^12,13^ The larger decrease in mitochondrial bioenergetics in the hippocampus compared with the neocortex, therefore, was unexpected. The decrease in the hippocampal energetic capacity did at least temporally correlate with hippocampal dependent learning and memory impairments in the brain-injured mice in agreement with previous studies from our group assessing mitochondrial function in the basal ganglia and its correlation to motor function both in TBI and aging.^40,41^ We tested the possibility that gliosis in the cortex was masking the neuronal decrease in bioenergetics. However, our results do not support this argument because both synaptic and nonsynaptic mitochondria were found to have decreased respiration.

There are two additional explanations for the selective vulnerability of the hippocampal mitochondria to TBI. The first possibility is that neurons in the hippocampus are more sensitive to the damaging effects of oxidative stress, and this resulted in increased damage to the mitochondria. The second possibility is that biomechanical forces of the CHI affected the hippocampus more than the cortex. Studies are ongoing to test these two possibilities.

Head injuries are strongly correlated with cognitive deterioration and neurodegenerative dementia, including Alzheimer disease.^42^ It is assumed widely that the effects of a mild TBI, such as dizziness and headaches, are temporary. Evidence suggests that this is not the case, and even a mild TBI can result in progressive brain atrophy.^43^ All blows to the head do not lead to neurodegeneration; however, they do increase the likelihood that dementia may develop in the person's lifetime. Our work highlights a potential mechanism by which a mild TBI could lead to cognitive decline through a process of premature mitochondrial aging. Therefore, we sought to test translatable biomarkers that could be used to identify patients with mild TBI who are showing signs of TBI-induced mitochondrial aging.

### Advanced neuroimaging is useful at detecting changes in surrogate markers brain metabolism and neuronal health that are altered after a mild TBI

Recent positive acute stroke intervention clinical trials highlight the importance of using advanced neuroimaging-based selection criteria in the successful outcomes of the trials.^44^ The Stroke Imaging Research (STIR) group has identified that: (i) Neuroimaging can be used as an efficacy/biomarker or as an outcome assessment in clinical trials; (ii) no one single imaging approach will address all clinical requirements; (iii) use of imaging outcomes as a restrictive selection criterion may reduce the heterogeneity in the study population allowing for studies with smaller sample size requirements.^45^ The guidelines outline by the STIR group are exceedingly relevant for mild TBI, because the number of cases of mild TBI are numerous, but the percentage of cases that will have clinical difficulties associated with the mild TBI represent a small proportion of the total number of cases (15%).^46,47^

A primary goal of our study was to reverse translate two advanced neuroimaging surrogate markers of brain metabolism, which have shown utility clinically in mild TBI ^7–9^ to a mouse model of mild TBI. We chose to use MR methods instead of positron emission tomography (PET) to measure brain metabolism, because of the wider availability of MR scanners in nonacademic clinical centers, and the advantage of not exposing persons to potentially toxic PET ligands. Our two primary MR methods used were pCASL to measure CBF and ^1^H-MRS to measure hippocampal metabolites.

In agreement with previous human studies,^48–51^ we were able to detect changes in CBF in mice after a mild TBI. The pCASL sequence used in this study has greater specificity in analyzing CBF than other common methods.^21,27^ We found substantial amounts of variability in all groups, however. Because we found that a TBI caused regionally based changes in CBF, with some areas showing hypoperfusion and adjacent areas showing hyperperfusion, it was necessary to use small ROIs. Despite the increased sensitivity of the 7-Tesla scanner and the pCASL sequence, we believe that the small ROIs used in the study were at least in part associated with the substantial variability in CBF seen between animals. In support of our assumption that the size of the ROI was at least partially associated with the increased variability, we found the lowest within group variance in the thalamus and hippocampus, which had the largest ROIs.

Improved MR sequences and scanners hold the potential to overcome the spatial limitations in the ability to define regional variations in CBF in mice after a mild CHI. This is only a limitation for pre-clinical studies, however. While we only see a trend in our mouse models after TBI, the results are nevertheless promising that pCASL could be useful in humans or larger animal models of TBI to identify metabolic disturbances caused by mild TBI.

The second neuroradiological method used to measure metabolic disturbances was ^1^H-MRS. In our pilot experiment, we identified three hippocampal metabolites that were significantly decreased from sham levels, namely PCr, NAA, and tCHo. In a second large cohort of mice, we used a more sensitive ^1^H-MRS method to validate these three metabolite changes and determine the potential of ^1^H-MRS to be a surrogate indicator of brain metabolism and neuronal health. PCr is directly associated with mitochondrial energy metabolism and is used as a source of energy to maintain ATP when levels of ATP are limited.^52^ In agreement with the decreases in mitochondrial respiration found in the hippocampus at 28 days after the brain injury, we also found decreases in PCr in the hippocampus at 28 days after injury by ^1^H-MRS.

Our data are in accordance with a recent study in athletes who were exposed to a mild brain injury.^53^ We found that a significant proportion of the head-injured mice decreased PCr below all sham-injured mice, suggesting that PCr levels accurately define injured versus uninjured animals with metabolic disturbances. In addition, we found that NAA, a marker for neuronal viability, which has been observed to decrease in patients in the acute phases after mild TBI,^8, 54^ was also able to accurately define a subset of injured versus uninjured animals.

In contrast, while we found a statistically significant decrease in tCho levels at three days post-injury, tCho values were not able to accurately separate injured from uninjured animals. In agreement with our findings, the peak decrease in tCho after a moderate TBI in rats occurred three days post-injury, after which point the levels of tCho began to rise.^55^ Changes in tCho have been reported in persons after a mild to moderate head injury; however, the levels of tCho were found to be increased after injury.^54,56^ Similarly, we found that tCho were slightly elevated over sham values at 28 days post-injury. The results suggest a dynamic pattern of tCho values depending on the time after injury when measurements are made. The temporal changes and limited diagnostic potential limits the usefulness of tCho as an efficacy biomarker of intervention studies. The changes in hippocampal metabolites reported in the current study are in contrast to those of a recent study in a rat model of mild TBI, which failed to find differences in cortical metabolites after the injury.^57^ Differences between the two studies are numerous and include species, TBI model, brain region, sample size, and spectroscopy method. We believe that the higher field strength scanner, improved water suppression sequence,^20^ and the larger sample size used in the current study were all important for our ability to detect changes in brain metabolites after the mild TBI.

### Limitations of the current study

Our studies included an equal proportion of female and male mice. Most of our end points were not sufficiently statistically powered to be disaggregated and reported by sex. The sample size available for the ^1^H-MRS study was adequate to stratify the data by sex for exploratory analysis. For PCr, NAA, and tCho, we found a similar pattern of CHI-induced changes between the female and male mice; however, the CHI-induced changes in the metabolites did not reach statistical significance in the male mice, likely because of decreased statistical power with the smaller sample size. Interestingly, the exploratory analysis found that GABA, lactate, and myoinositol were decreased by the CHI in the female mice only. Future studies will be needed to determine how sexually dimorphic characteristics, chromosomal differences, and sex hormone levels may contribute to the sex-specific effects on GABA, lactate, and myoinositol.

Multi-modal MR is noninvasive, but in mice, it does require extended periods of anesthesia, with a range of 30–90 min, depending whether one or two sequences are acquired. Using isoflurane, we were able to regulate the depth of the anesthesia to maintain respiration. In addition, each imaging session required the mice to be under anesthesia. Studies have shown that isoflurane can be protective after injury.^58^ Other studies have also demonstrated that prolonged isoflurane can impact CBF and mitochondrial function.^59^ There is an advantage to repetitive scans in the same mouse, including establishing a pre-injury baseline. We do not know the effect that multiple rounds of extended anesthesia have on the recovery of TBI and brain metabolite levels. Therefore, we did not attempt to establish the time course of MR changes in the same mouse. For these same reasons, we used separate cohorts of mice for our mitochondrial bioenergetics measurements.

## Conclusion

In this report, we find that a mild TBI results in a mitochondrial bioenergetics response that is akin to accelerated aging. We found a regional heterogeneity in mitochondrial bioenergetics that suggests selective vulnerability of neurons in different regions of the brain to the effect of TBI. Finally, we validated ^1^H-MRS as a surrogate indicator of bioenergetic and neuronal health after a mild TBI.

## References

[1] Sullivan P.G. and Brown M.R. (2005). Mitochondrial aging and dysfunction in Alzheimer's disease. Prog. Neuropsychopharmacol. Biol. Psychiatry 29, 407–410 15795049 10.1016/j.pnpbp.2004.12.007

[2] Yonutas H.M., Vekaria H.J., and Sullivan P.G. (2016). Mitochondrial specific therapeutic targets following brain injury. Brain Res. 1640, 77–93 26872596 10.1016/j.brainres.2016.02.007

[3] Brown M.R., Sullivan P.G., and Geddes J.W. (2006). Synaptic mitochondria are more susceptible to Ca2+ overload than nonsynaptic mitochondria. J. Biol. Chem. 281, 11658–11668 16517608 10.1074/jbc.M510303200

[4] Barnes D.E., Kaup A., Kirby K.A., Byers A.L., Diaz-Arrastia R., and Yaffe K. (2014). Traumatic brain injury and risk of dementia in older veterans. Neurology. 83, 312–319 24966406 10.1212/WNL.0000000000000616 PMC4115602

[5] Johnson V.E., Stewart W., and Smith D.H. (2010). Traumatic brain injury and amyloid-beta pathology: a link to Alzheimer's disease? Nat. Rev. Neurosci. 11, 361–370 20216546 10.1038/nrn2808 PMC3979339

[6] Wu X., Kirov II, Gonen O., Ge Y.L., Grossman R.I., and Lui Y.W. (2016). MR imaging applications in mild traumatic brain injury: an imaging update. Radiology 279, 693–707 27183405 10.1148/radiol.16142535 PMC4886705

[7] Wang Y., Nelson L.D., LaRoche A.A., Pfaller A.Y., Nencka A.S., Koch K.M., and McCrea M.A. (2016). Cerebral blood flow alterations in acute sport-related concussion. J. Neurotrauma 33, 1227–1236 26414315 10.1089/neu.2015.4072 PMC4931342

[8] George E.O., Roys S., Sours C., Rosenberg J., Zhuo J., Shanmuganathan K., and Gullapalli R.P. (2014). Longitudinal and prognostic evaluation of mild traumatic brain injury: a 1H-magnetic resonance spectroscopy study. J. Neurotrauma. 31, 1018–1028 24467391 10.1089/neu.2013.3224

[9] Maudsley A.A., Govind V., Levin B., Saigal G., Harris L., and Sheriff S. (2015). Distributions of magnetic resonance diffusion and spectroscopy measures with traumatic brain injury. J. Neurotrauma 32, 1056–1063 25333480 10.1089/neu.2014.3505 PMC4504344

[10] Vagnozzi R., Signoretti S., Cristofori L., Alessandrini F., Floris R., Isgro E., Ria A., Marziali S., Zoccatelli G., Tavazzi B., Del Bolgia F., Sorge R., Broglio S.P., McIntosh T.K., and Lazzarino G. (2010). Assessment of metabolic brain damage and recovery following mild traumatic brain injury: a multicentre, proton magnetic resonance spectroscopic study in concussed patients. Brain 133, 3232–3242 20736189 10.1093/brain/awq200

[11] Vagnozzi R., Signoretti S., Tavazzi B., Floris R., Ludovici A., Marziali S., Tarascio G., Amorini A.M., Di Pietro V., Delfini R., and Lazzarino G. (2008). Temporal window of metabolic brain vulnerability to concussion: a pilot 1H-magnetic resonance spectroscopic study in concussed athletes—part III. Neurosurgery 62, 1286–1295 18824995 10.1227/01.neu.0000333300.34189.74

[12] Bachstetter A.D., Webster S.J., Goulding D.S., Morton J.E., Watterson D.M., and Van Eldik L.J. (2015). Attenuation of traumatic brain injury-induced cognitive impairment in mice by targeting increased cytokine levels with a small molecule experimental therapeutic. J. Neuroinflammation 12, 69 25886256 10.1186/s12974-015-0289-5 PMC4396836

[13] Webster S.J., Van Eldik L.J., Watterson D.M., and Bachstetter A.D. (2015). Closed head injury in an age-related Alzheimer mouse model leads to an altered neuroinflammatory response and persistent cognitive impairment. J. Neurosci. 35, 6554–6569 25904805 10.1523/JNEUROSCI.0291-15.2015 PMC4405562

[14] Brody D.L., Mac Donald C., Kessens C.C., Yuede C., Parsadanian M., Spinner M., Kim E., Schwetye K.E., Holtzman D.M., and Bayly P.V. (2007). Electromagnetic controlled cortical impact device for precise, graded experimental traumatic brain injury. J. Neurotrauma 24, 657–673 17439349 10.1089/neu.2006.0011 PMC2435168

[15] Pandya J.D., Readnower R.D., Patel S.P., Yonutas H.M., Pauly J.R., Goldstein G.A., Rabchevsky A.G., and Sullivan P.G. (2014). N-acetylcysteine amide confers neuroprotection, improves bioenergetics and behavioral outcome following TBI. Exp. Neurol. 257, 106–113 24792639 10.1016/j.expneurol.2014.04.020 PMC4086163

[16] Sauerbeck A., Pandya J., Singh I., Bittman K., Readnower R., Bing G., and Sullivan P. (2011). Analysis of regional brain mitochondrial bioenergetics and susceptibility to mitochondrial inhibition utilizing a microplate based system. J. Neurosci. Methods. 198, 36–43 21402103 10.1016/j.jneumeth.2011.03.007 PMC3535268

[17] Alamed J., Wilcock D.M., Diamond D.M., Gordon M.N., and Morgan D. (2006). Two-day radial-arm water maze learning and memory task; robust resolution of amyloid-related memory deficits in transgenic mice. Nat. Protoc. 1, 1671–1679 17487150 10.1038/nprot.2006.275

[18] Guo J., Bakshi V., and Lin A.L. (2015). Early shifts of brain metabolism by caloric restriction preserve white matter integrity and long-term memory in aging mice. Front. Aging Neurosci. 7, 213 26617514 10.3389/fnagi.2015.00213 PMC4643125

[19] Parikh I., Guo J., Chuang K.H., Zhong Y., Rempe R.G., Hoffman J.D., Armstrong R., Bauer B., Hartz A.M., and Lin A.L. (2016). Caloric restriction preserves memory and reduces anxiety of aging mice with early enhancement of neurovascular functions. Aging (Albany NY). 8, 2814–2826 27829242 10.18632/aging.101094 PMC5191872

[20] Deelchand D.K., Adanyeguh I.M., Emir U.E., Nguyen T.M., Valabregue R., Henry P.G., Mochel F., and Oz G. (2015). Two-site reproducibility of cerebellar and brainstem neurochemical profiles with short-echo, single-voxel MRS at 3T. Magn. Reson. Med. 73, 1718–1725 24948590 10.1002/mrm.25295 PMC4272339

[21] Alsop D.C., Detre J.A., Golay X., Gunther M., Hendrikse J., Hernandez-Garcia L., Lu H., MacIntosh B.J., Parkes L.M., Smits M., van Osch M.J., Wang D.J., Wong E.C., and Zaharchuk G. (2015). Recommended implementation of arterial spin-labeled perfusion MRI for clinical applications: A consensus of the ISMRM perfusion study group and the European consortium for ASL in dementia. Magn. Reson. Med. 73, 102–116 24715426 10.1002/mrm.25197 PMC4190138

[22] Provencher S.W. (1993). Estimation of metabolite concentrations from localized in vivo proton NMR spectra. Magn. Reson. Med. 30, 672–679 8139448 10.1002/mrm.1910300604

[23] Oz G., Nelson C.D., Koski D.M., Henry P.G., Marjanska M., Deelchand D.K., Shanley R., Eberly L.E., Orr H.T. and Clark H.B. (2010). Noninvasive detection of presymptomatic and progressive neurodegeneration in a mouse model of spinocerebellar ataxia type 1. J Neurosci. 30, 3831-8 20220018 10.1523/JNEUROSCI.5612-09.2010 PMC2846771

[24] Graaf R.A. In vivo NMR Spectroscopy: Principles and Techniques. Chichester: John Wiley & Sons, Ltd., 2007

[25] CDC. (2003). Report to Congress on mild traumatic brain injury in the United States: steps to prevent a serious public health problem. Atlanta, GA: US Department of Health and Human Services. CDC

[26] Faul M., Xu L., Wald M.M. and Coronado V.G. (2010). Traumatic brain injury in the United States: emergency department visits, hospitalizations, and deaths. Atlanta (GA): Centers for Disease Control and Prevention, National Center for Injury Prevention and Control

[27] Hong X., To X.V., The I., Soh J.R., and Chuang K.H. (2015). Evaluation of EPI distortion correction methods for quantitative MRI of the brain at high magnetic field. Magn. Reson. Imaging. 33, 1098–1105 26117700 10.1016/j.mri.2015.06.010

[28] Adam O., Mac Donald C.L., Rivet D., Ritter J., May T., Barefield M., Duckworth J., LaBarge D., Asher D., Drinkwine B., Woods Y., Connor M., and Brody D.L. (2015). Clinical and imaging assessment of acute combat mild traumatic brain injury in Afghanistan. Neurology. 85, 219–227 26109715 10.1212/WNL.0000000000001758 PMC4516289

[29] Janowitz T. and Menon D.K. (2010). Exploring new routes for neuroprotective drug development in traumatic brain injury. Sci. Transl. Med. 2, 27rv1 10.1126/scitranslmed.3000330 20393189

[30] Pandya J.D., Royland J.E., MacPhail R.C., Sullivan P.G., and Kodavanti P.R. (2016). Age- and brain region-specific differences in mitochondrial bioenergetics in Brown Norway rats. Neurobiol. Aging 42, 25–34 27143418 10.1016/j.neurobiolaging.2016.02.027

[31] Goyal M.S., Vlassenko A.G., Blazey T.M., Su Y., Couture L.E., Durbin T.J., Bateman R.J., Benzinger T.L., Morris J.C., and Raichle M.E. (2017). Loss of brain aerobic glycolysis in normal human aging. Cell Metab. 26, 353–360e3 28768174 10.1016/j.cmet.2017.07.010 PMC5573225

[32] Harman D. (1956). Aging: a theory based on free radical and radiation chemistry. J. Gerontol. 11, 298–300 13332224 10.1093/geronj/11.3.298

[33] Balaban R.S., Nemoto S., and Finkel T. (2005). Mitochondria, oxidants, and aging. Cell 120, 483–495 15734681 10.1016/j.cell.2005.02.001

[34] Pandya J.D., Nukala V.N., and Sullivan P.G (2013). Concentration dependent effect of calcium on brain mitochondrial bioenergetics and oxidative stress parameters. Front. Neuroenergetics 5, 10 24385963 10.3389/fnene.2013.00010 PMC3866544

[35] Opii W.O., Nukala V.N., Sultana R., Pandya J.D., Day K.M., Merchant M.L., Klein J.B., Sullivan P.G., and Butterfield D.A. (2007). Proteomic identification of oxidized mitochondrial proteins following experimental traumatic brain injury. J. Neurotrauma 24, 772–789 17518533 10.1089/neu.2006.0229

[36] Singh I.N., Sullivan P.G., Deng Y., Mbye L.H., and Hall E.D. (2006). Time course of post-traumatic mitochondrial oxidative damage and dysfunction in a mouse model of focal traumatic brain injury: implications for neuroprotective therapy. J. Cereb. Blood Flow Metab. 26, 1407–1418 16538231 10.1038/sj.jcbfm.9600297

[37] Wang X. and Michaelis E.K. (2010). Selective neuronal vulnerability to oxidative stress in the brain. Front. Aging Neurosci. 2, 12 20552050 10.3389/fnagi.2010.00012 PMC2874397

[38] Kulbe J.R., Hill R.L., Singh I.N., Wang J.A., and Hall E.D. (2017). Synaptic mitochondria sustain more damage than non-synaptic mitochondria after traumatic brain injury and are protected by cyclosporine A. J. Neurotrauma 34, 1291–1301 27596283 10.1089/neu.2016.4628 PMC5385586

[39] Sullivan P.G., Keller J.N., Mattson M.P., and Scheff S.W. (1998). Traumatic brain injury alters synaptic homeostasis: implications for impaired mitochondrial and transport function. J. Neurotrauma 15, 789–798 9814635 10.1089/neu.1998.15.789

[40] Pandya J.D., Grondin R., Yonutas H.M., Haghnazar H., Gash D.M., Zhang Z., and Sullivan P.G. (2015). Decreased mitochondrial bioenergetics and calcium buffering capacity in the basal ganglia correlates with motor deficits in a nonhuman primate model of aging. Neurobiol. Aging 36, 1903–1913 25726361 10.1016/j.neurobiolaging.2015.01.018

[41] Sauerbeck A., Hunter R., Bing G., and Sullivan P.G. (2012). Traumatic brain injury and trichloroethylene exposure interact and produce functional, histological, and mitochondrial deficits. Exp. Neurol. 234, 85–94 22201550 10.1016/j.expneurol.2011.12.012 PMC3294257

[42] Gardner R.C. and Yaffe K. (2015). Epidemiology of mild traumatic brain injury and neurodegenerative disease. Mol. Cell Neurosci. 66, 75–80 25748121 10.1016/j.mcn.2015.03.001 PMC4461453

[43] Zhou Y., Kierans A., Kenul D., Ge Y., Rath J., Reaume J., Grossman R.I., and Lui Y.W. (2013). Mild traumatic brain injury: longitudinal regional brain volume changes. Radiology 267, 880–890 23481161 10.1148/radiol.13122542 PMC3662902

[44] Warach S.J., Luby M., Albers G.W., Bammer R., Bivard A., Campbell B.C., Derdeyn C., Hei t J.J., Khatri P., Lansberg M.G., Liebeskind D.S., Majoie C., Marks M.P., Menon B.K., Muir K.W., Parsons M.W., Vagal A., Yoo A.J., Alexandrov A.V., Baron J.C., Fiorella D.J., Furlan A.J., Puig J., Schellinger P.D., Wintermark M., Stroke Imaging Research (STIR) and VISTA Imaging Investigators. (2016). Acute stroke imaging research roadmap III imaging selection and outcomes in acute stroke reperfusion clinical trials consensus recommendations and further research priorities. Stroke 47, 1389–1398 27073243 10.1161/STROKEAHA.115.012364 PMC6058693

[45] Wintermark M., Albers G.W., Broderick J.P., Demchuk A.M., Fiebach J.B., Fiehler J., Grotta J.C., Houser G., Jovin T.G., Lees K.R., Lev M.H., Liebeskind D.S., Luby M., Muir K.W., Parsons M.W., von Kummer R., Wardlaw J.M., Wu O., Yoo A.J., Alexandrov A.V., Alger J.R., Aviv R.I., Bammer R., Baron J.C., Calamante F., Campbell B.C., Carpenter T.C., Christensen S., Copen W.A., Derdeyn C.P., Haley C., Khatri P., Kudo K., Lansberg M.G., Latour L.L., Lee T.Y., Leigh R., Lin W.L., Lyden P., Mair G., Menon B.K., Michel P., Mikulik R., Nogueira R.G., Ostergaard L., Pedraza S., Riedel C.H., Rowley H.A., Sanelli P.C., Sasaki M., Saver J.L., Schaefer P.W., Schellinger P.D., Tsivgoulis G., Wechsler L.R., White P.M., Zaharchuk G., Zaidat O.O., Davis S.M., Donnan G.A., Furlan A.J., Hacke W., Kang D.W., Kidwell C., Thijs V.N., Thomalla G., Warach S.J., Stroke Imaging Research (STIR) and Virtual Int ernational Stroke Trials Archive (VISTA)- Imaging Investigators (2013). Acute Stroke Imaging Research Roadmap II. Stroke 44, 2628–2639 23860298 10.1161/STROKEAHA.113.002015 PMC4040226

[46] Roe C., Sveen U., Alvsaker K., and Bautz-Holter E. (2009). Post-concussion symptoms after mild traumatic brain injury: influence of demographic factors and injury severity in a 1-year cohort study. Disabil. Rehabil. 31, 1235–1243 19116810 10.1080/09638280802532720

[47] Williams W.H., Potter S., and Ryland H. (2010). Mild traumatic brain injury and Postconcussion Syndrome: a neuropsychological perspective. J. Neurol. Neurosurg. Psychiatry 81, 1116–1122 20802217 10.1136/jnnp.2008.171298

[48] Martin N.A., Patwardhan R.V., Alexander M.J., Africk C.Z., Lee J.H., Shalmon E., Hovda D.A., and Becker D.P. (1997). Characterization of cerebral hemodynamic phases following severe head trauma: hypoperfusion, hyperemia, and vasospasm. J. Neurosurg. 87, 9–19 9202259 10.3171/jns.1997.87.1.0009

[49] Lin C.M., Tseng Y.C., Hsu H.L., Chen C.J., Chen D.Y., Yan F.X., and Chiu W.T. (2016). Arterial spin labeling perfusion study in the patients with subacute mild traumatic brain injury. PLoS One 11, e0149109 26871696 10.1371/journal.pone.0149109 PMC4752493

[50] Bouzat P., Almeras L., Manhes P., Sanders L., Levrat A., David J.S., Cinotti R., Chabanne R., Gloaguen A., Bobbia X., Thoret S., Oujamaa L., Bosson J.L., Payen J.F., Asehnoune K., Pes P., Lefrant J.Y., Mirek S., Albasini F., Scrimgeour C., Thouret J.M., Chartier F., Ginet M., and TBI-TCD Study Investigators. (2016). Transcranial Doppler to predict neurologic outcome after mild to moderate traumatic brain injury. Anesthesiology 125, 346–354 27224640 10.1097/ALN.0000000000001165

[51] Peng S.P., Li Y.N., Liu J., Wang Z.Y., Zhang Z.S., Zhou S.K., Tao F.X., and Zhang Z.X. (2016). Pulsed arterial spin labeling effectively and dynamically observes changes in cerebral blood flow after mild traumatic brain injury. Neural. Regen. Res. 11, 257–261 27073378 10.4103/1673-5374.177733 PMC4810989

[52] Schlattner U., Tokarska-Schlattner M., and Wallimann T. (2006). Mitochondrial creatine kinase in human health and disease. Biochim. Biophys. Acta 1762, 164–180 16236486 10.1016/j.bbadis.2005.09.004

[53] Poole V.N., Breedlove E.L., Shenk T.E., Abbas K., Robinson M.E., Leverenz L.J., Nauman E.A., Dydak U., and Talavage T.M. (2015). Sub-concussive hit characteristics predict deviant brain metabolism in football athletes. Dev. Neuropsychol. 40, 12–17 25649774 10.1080/87565641.2014.984810

[54] Govind V., Gold S., Kaliannan K., Saigal G., Falcone S., Arheart K.L., Harris L., Jagid J., and Maudsley A.A. (2010). Whole-brain proton MR spectroscopic imaging of mild-to-moderate traumatic brain injury and correlation with neuropsychological deficits. J. Neurotrauma 27, 483–496 20201668 10.1089/neu.2009.1159 PMC2867627

[55] Harris J.L., Yeh H.W., Choi I.Y., Lee P., Berman N.E., Swerdlow R.H., Craciunas S.C., and Brooks W.M. (2012). Altered neurochemical profile after traumatic brain injury: (1)H-MRS biomarkers of pathological mechanisms. J. Cereb. Blood Flow Metab. 32, 2122–2134 22892723 10.1038/jcbfm.2012.114 PMC3519407

[56] Govindaraju V., Gauger G.E., Manley G.T., Ebel A., Meeker M., and Maudsley A.A. (2004). Volumetric proton spectroscopic imaging of mild traumatic brain injury. AJNR Am. J. Neuroradiol. 25, 730–737 15140711 PMC7974501

[57] Wright D.K., Trezise J., Kamnaksh A., Bekdash R., Johnston L.A., Ordidge R., Semple B.D., Gardner A.J., Stanwell P., O'Brien T.J., Agoston D.V., and Shultz S.R. (2016). Behavioral, blood, and magnetic resonance imaging biomarkers of experimental mild traumatic brain injury. Sci. Rep. 6, 28713 27349514 10.1038/srep28713 PMC4923906

[58] Zhou Y., Lekic T., Fathali N., Ostrowski R.P., Martin R.D., Tang J., and Zhang J.H. (2010). Isoflurane posttreatment reduces neonatal hypoxic-ischemic brain injury in rats by the sphingosine-1-phosphate/phosphatidylinositol-3-kinase/Akt pathway. Stroke 41, 1521–1527 20508187 10.1161/STROKEAHA.110.583757 PMC2917259

[59] Sosunov S.A., Ameer X., Niatsetskaya Z.V., Utkina-Sosunova I., Ratner V.I., and Ten V.S. (2015). Isoflurane anesthesia initiated at the onset of reperfusion attenuates oxidative and hypoxic-ischemic brain injury. PLoS One 10, e0120456 25799166 10.1371/journal.pone.0120456 PMC4370491

